# Wavelength Selection FOR Rapid Identification of Different Particle Size Fractions of Milk Powder Using Hyperspectral Imaging

**DOI:** 10.3390/s20164645

**Published:** 2020-08-18

**Authors:** Asma Khan, Muhammad Tajammal Munir, Wei Yu, Brent Young

**Affiliations:** 1 Chemical and Materials Engineering Department, University of Auckland, Auckland 1010, New Zealand; akha273@aucklanduni.ac.nz (A.K.); w.yu@auckland.ac.nz (W.Y.); 2 College of Engineering and Technology, American University of the Middle East, Egaila 54200, Kuwait; Muhammad.Munir@aum.edu.kw

**Keywords:** milk powder, hyperspectral imaging, principal component analysis, weighted regression coefficients analysis

## Abstract

Hyperspectral imaging (HSI) in the spectral range of 400–1000 nm was tested to differentiate three different particle size fractions of milk powder. Partial least squares discriminant analysis (PLS-DA) was performed to observe the relationship of spectral data and particle size information for various samples of instant milk powder. The PLS-DA model on full wavelengths successfully classified the three fractions of milk powder with a coefficient of prediction 0.943. Principal component analysis (PCA) identified each of the milk powder fractions as separate clusters across the first two principal components (PC_1_ and PC_2_) and five characteristic wavelengths were recognised by the loading plot of the first three principal components. Weighted regression coefficient (WRC) analysis of the partial least squares model identified 11 important wavelengths. Simplified PLS-DA models were developed from two sets of reduced wavelengths selected by PCA and WRC and showed better performance with predictive correlation coefficients (R_p_^2^) of 0.962 and 0.979, respectively, while PLS-DA with complete spectrum had R_p_^2^ of 0.943. Similarly, classification accuracy of PLS-DA was improved to 92.2% for WRC based predictive model. Calculation time was also reduced to 2.1 and 2.8 s for PCA and WRC based simplified PLS-DA models in comparison to the complete spectrum model that was taking 32.2 s on average to predict the classification of milk powder samples. These results demonstrated that HSI with appropriate data analysis methods could become a potential analyser for non-invasive testing of milk powder in the future.

## 1. Introduction

Milk powder quality is complex as it is measured in terms of different quality attributes such as milk powder appearance, taste, aroma, and its dissolution performance in water. These quality attributes of milk powder depend on various physical (e.g., particle size distribution, bulk density) and functional properties (e.g., wettability, sinkability, dispersibility, and solubility) of milk powder. The physical properties determine the storage and transport properties of milk powder, and functional properties describe how well the milk powder performs when recombined. Mostly, milk powder quality is measured after the fact and offline using various quality tests which include quality test(s) for measuring powder bulk density, powder flowability and dispersibility in water, etc. These quality tests have inherent variability and lack numeric descriptors which makes milk powder quality quantification challenging. Furthermore, this offline strategy of testing milk powder quality is not helpful to detect milk powder quality in real-time which is described as Process Analytical Technology (PAT) by the U.S. Food and Drug Administration [1]. Consequently, manual offline quality testing of milk powder needs to be replaced by machine vision for quality testing, which is relatively faster, has less variability, and has numeric descriptors.

Process analysers such as hyperspectral imaging (HSI) may potentially be used for testing milk powder quality and replacing existing manual quality tests because HSI is non-invasive, relatively faster than manual quality tests, and it can test powder quality in terms of quantifiable numeric descriptors. Hyperspectral imaging is popular because it combines the advantages of conventional imaging and spectroscopy to achieve the benefits of both techniques. During HSI data acquisition process, HSI instrument captures two-dimensional spatial (*x*, *y*) images at different wavelengths which is also called the spectral (*λ*) range. As a result, three-dimensional hypercube is obtained. Further details of HSI image acquisition and hypercube analysis are given by Amigo [2]. It is worth mentioning that imaging and spectroscopy are acknowledged techniques in the food industry. For example, in the food industry, imaging is used for visual defect detection [3], and spectroscopy is used for compositional analysis of food products and the identification of adulterants [4]. Hyperspectral imaging has been previously used in the food industry and other applications such as remote sensing, airborne surveys, astronomy, agriculture, biomedical, mineralogy, and pharmaceuticals [5]. In the food industry, HSI has been used for qualitative assessment of products, for example, monitoring the freshness and quality of meat [6], identifying the types and varieties of cereals [7], detecting defects in fruits and vegetables [8], and exploring varieties of cheese and its quality [9].

The hypercube obtained after data acquisition needs appropriate multivariate data analysis tools that can relate the hyperspectral data with milk powder quality attributes. Principal component analysis (PCA), discriminant analysis (DA), and partial least square (PLS) regression are among the common multivariate data analysis tools reported in the literature which can be used to explore the relationship of spectral variation with the chemical/physical/functional properties [10,11]. Furthermore, suitable data pre-processing techniques such as normalisation, de-noising, smoothing, filtering, and/or taking derivatives of the spectra are required for preparing data sets for subsequent data analysis.

Hyperspectral imaging usually generates large data sets of information. Nevertheless, there might be redundancy of information in the consecutive bands of HSI data. These highly correlated wavelengths of similar information could affect the performance of multivariate data analysis [12]. Hyperspectral imaging data analysis can be utilised in real-world applications if vital wavelengths can be identified. However, one struggles to find standard selection criteria for obtaining the important wavelengths from the full spectrum. Several key wavelengths are usually recognised through more than one strategy such as PCA [13], weighted regression coefficient (WRC) analysis [14], successive projection algorithm (SPA) [15], uninformative variable elimination (UVE) [16], and stepwise regression coefficient analysis [17]. This reduction in the number of selected wavelengths is called feature selection in multivariate data analysis. Selected wavelengths are used to propose a reliable multispectral imaging system that could represent the complete spectrum well. The accuracy and fast acquisition of the results are important considerations for the development of real-time quality monitoring systems. On this basis, the selection of reduced wavelengths is essential for reducing the amount of data acquisition and processing for an HSI application.

In previous studies HSI has been used for detection of adulterants (such as, melamine, urea, etc.) in milk powders [18,19,20]. Two important wavelengths 1447 nm and 1466 nm are reported to detect melamine by spectral analysis. Melamine has low reflectance ability in the region 1450–1550 nm and band ratio technique was used for the spectral analysis of milk powder and melamine [21]. Milk powders of varying quality produced at different production locations were discriminated by HSI [22]. However, there is no reported example for identifying vital wavelengths for estimating the milk powders quality attributes (either physical or functional properties) by HSI.

This paper aims to evaluate the potential of HSI techniques to facilitate the rapid testing of milk powder. This article has three main specific objectives. The first objective was to develop a partial least squares discriminant analysis (PLS-DA) model to differentiate and classify milk powders according to the varying size range of particles, because the published literature has shown that powder particle size and particle size distribution are influential to functional performance (rehydration characteristics) and quality of milk powder [23,24]. The second objective was to identify the important wavelengths by PCA and WRC methods because data reduction to a manageable size is required for real-world applications. The last objective was to develop simplified PLS-DA models with a reduced number of wavelengths to classify the particle size fractions of milk powder which can be implemented for real-time quality monitoring in the future.

## 2. Materials and Methods

### 2.1. Milk Powder Sample Preparation

Ten different batches of commercial grade milk powders of a locally manufactured brand were purchased from the supermarket. A Retsch AS200 vibratory sieve shaker was used with two sieves of 180 µm and 355 µm aperture to segregate the milk powder into three discrete particle size fractions: coarse particles fraction (labelled as ‘C’, having particle diameter larger than 355 μm), medium particles fraction (labelled as ‘M’, having particle diameter larger than 180 μm and smaller than 355 μm), and fine particles fractions (labelled as ‘F’, having particle diameter smaller than 180 μm). Three sets of each particle size fraction were prepared from a single batch and a total of 30 samples of each particle fraction were used in further analysis.

A recombined sample of milk powder was prepared with 20% (wt./wt.) fines particle fraction, 60% (wt./wt.) medium particle fraction and 20% (wt./wt.) coarse particle fraction. This recombined sample was used for visualisation of classification results only.

### 2.2. Hyperspectral Imaging Setup

**Figure 1:**
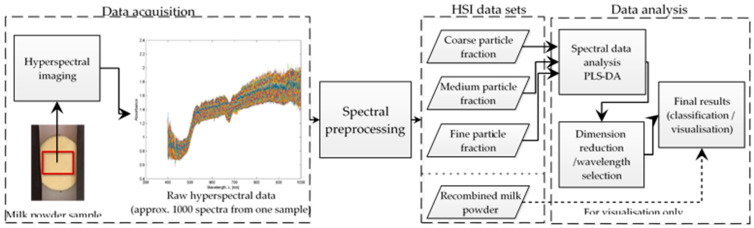
Flowchart of the key steps involved in hyperspectral imaging analysis.

In this study, we employed a Headwall Photonics Hyperspec^TM^ VNIR HSI instrument. This HSI instrument had a sensor covering VNIR (visible and near-infrared) wavelengths in the range from 400–1000 nm. The HSI instrument consists of four basic components: a spectrograph, camera (Schneider-Kreuznach Xenoplan 1.4/23), lamp, and transition stage. The camera captured spatial images of samples that had pixels, and each pixel represented a spectrum through the images. The lamp provided a lighting source, and the transition stage provided a moveable platform for placing samples for the analysis. The HSI equipment was enclosed in a black box while analysing the samples to minimise the impact of ambient light. Hyperspectral imaging analysis involves various steps involving data acquisition, image calibration, spectra pre-processing, and data analysis [10]. A flowchart is presented in Figure 1 that overviews these steps performed in this research.

Reflectance calibration was performed on all the images recorded by the hyperspectral equipment. A white reference image (*W*) was recorded from a standard Teflon tile provided by the manufacturer as an accessory with the equipment. A dark image (*D*) was saved as a response of the camera in the absence of light. The corrected image (*I*) of each sample was obtained from the respective recorded image (*I_sample_*) by Equation (1) and saved as a hypercube.

(1) $$I=\;\frac{I_{sample}-D}{W-D}$$

Three hyperspectral images of every sample of milk powder were recorded. Matlab R2018 (The MathWorks Inc., Natick, MA, USA) with the PLS Toolbox (Eigenvector Research, Inc., Manson, WA, USA) and the Unscrambler HSI (Camo Analytics AS, Oslo, Norway) were used for processing and analysis of the hypercube generated by the HSI.

### 2.3. Data Pre-Processing

**Figure 2:**
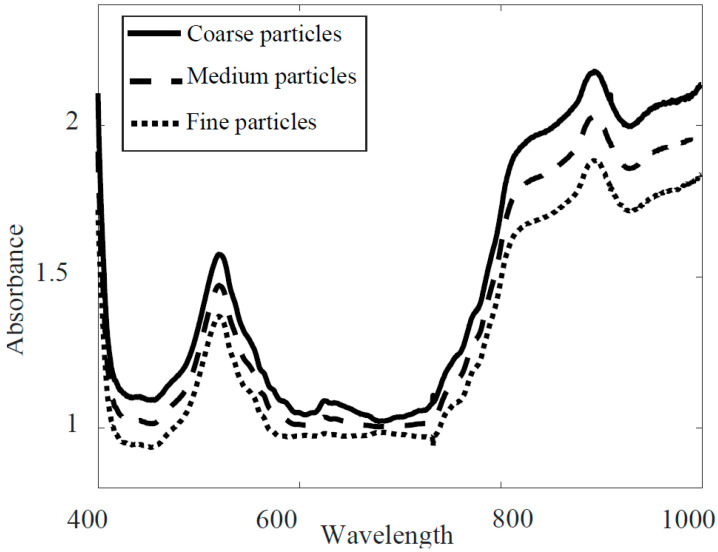
Average spectra of three different milk powder size fractions after pre-processing.

A square region of interest (ROI) with the same spatial resolution was manually selected for all the images. The standard normal variance (SNV) [25] was used for spectral data normalisation for each image of the milk powder sample. The HSI data was noisy when plotted as a function of wavelength. The impact of different pre-processing methods on the milk powder spectrum is discussed in a previous study [26]. Therefore, spectral smoothing was implemented by the Matlab function *smoothn* developed by Garcia [27]. This method was preferred for accommodating multi-dimensional data and providing robust smoothing of spectra generated by HSI. Earlier, Munir Wilson [22] reported this pre-processing method for HSI data of milk powders of varying quality obtained from different production locations. Average spectra of three discrete particle size fractions after preprocessing are presented in Figure 2, and show clear offset.

### 2.4. Multivariate Data Analysis

#### Partial Least Square Discriminant Analysis (PLS-DA)

Partial least square discriminant analysis (PLS-DA) is a supervised classification technique [28]. One group is assigned as variable 0 and a second group is assigned as variable 1. Prediction samples either belonging to variable/group 0 or 1 are classified as respective groups [29]. However, in this research there were three different particle size fractions of milk powder to be discriminated. We had spectra of each HSI image as a predictor matrix **X**, which was a function of **Y**, a variable set of assigned dummy values of 1, 2, 3 which were a respective reference to class C, M, and F. The parsimonious number of latent variables (LVs) from PLS analysis was determined by analysing the root mean square error of cross-validation (RMSECV).

Validation of the model is an important step in any data analysis. It provides the comparison of output provided by the model to the actual variable measured and has a significant impact on the reliability of the model. The samples were divided into calibration and prediction sets. Each particle size fraction had 30 samples prepared from 10 different batches. A total of 21 samples from seven batches were kept for calibrating the model. Whereas nine samples from three batches were used in the prediction models. A classification model was developed from the multi-pixel spectra extracted from each hyperspectral image of the coarse, medium, and fine milk powder samples. However, PLS-DA classification primarily performs regression between spectra and class membership [30]. Therefore, the performance of the calibration model was estimated by the correlation coefficient (R_c_^2^) and root mean square error of calibration (RMSEC). Cross-validation of the calibration model was used for internal validation of the model by the correlation coefficient of cross-validation (R_cv_^2^) and RMSECV, respectively. The prediction performance was also measured in terms of correlation coefficient of prediction (R_p_^2^) and root mean square error of prediction (RMSEP). For a good performance of the model correlation coefficients terms were expected to be close to 0.9 while the root mean square error terms should be close to zero [31]. Residual predictive deviation (RPD) was also calculated for the model. This is the ratio of the standard deviation of the calibration set to the sum of prediction errors. It is believed that a good model performance is associated with a high value of RPD. In general a RPD value greater than three is acceptable [31,32]. Confusion matrices for the prediction of coarse, medium, and fine particle fractions were also created. These confusion matrices show the true positive prediction rate for each particle fraction. Accuracy, sensitivity, and specificity of the classification was also determined from these confusion matrices.

Hyperspectral imaging data has the advantage of producing distribution maps for better visualization over traditional spectroscopic techniques. Presence of fine particles has a significant impact on the quality of the milk powder [33]. Therefore, chemical images and a distribution map were produced by pixel spectra of milk powder sample and the regression coefficient of a model.

### 2.5. Wavelength Selection

Accuracy and speed are required for HSI application in industrial settings. It would be expedient to use the big data generated from the HSI directly. However, data analysis based upon the full spectral range of 400–1000 nm could be affected by the collinearity of the similar spectral information of the consecutive wavebands. This high dimensionality of the HSI data has its impact on the computation speed and it could make data processing a time-consuming step. However, the data acquisition and its processing could be made more efficient and robust if optimum wavelengths that carry valuable information were identified.

#### 2.5.1. Principal Component Analysis (PCA)

Principal component analysis (PCA) is a well-known technique for dimension reduction in big data systems. PCA gives an overview of the data set on a new axis called the principal components (PCs). PCA extracts the systematic variation of data by projecting it into a new space across these PCs [34]. This technique was applied to the spectral data of the samples of three milk powder fractions from the seven batches assigned to calibration. It transformed the data in such a way that the projections of the transformed data (termed as the principal components) exhibit maximal variance among three fractions of milk powder. This data transformation was represented in the score plots of PCA. Three PCs were retained that represented the 75% variance of the data.

Principal component analysis is also one of the most extensively used feature selection methods. A band prioritisation method based on the PCA can be found in published domain of literature [13]. The loading plot represents the influence of wavelengths on the PCs. The influential wavelengths were extracted from local minima or maxima of the retained PCs. A similar approach was used for identifying six key wavelengths for apple bruise detection from HSI [35]. Influential wavelengths by three PC loadings were recognised to classify plastic and cotton using HSI technology [36].

#### 2.5.2. Weighted Regression Coefficient (WRC) Analysis

This method was based upon the regression coefficient analysis of the wavelengths obtained from the partial least squares model as shown in Equation (2).

**Y** = β**X′** (2)

Whereas $X^{\prime }$ was the standardised wavelength matrix obtained by dividing wavelength vectors by their standard deviation and Y was the predictor matrix. Both $X^{\prime }$ and Y were related by a regression vector β. The weighted regression coefficient (WRC) method was performed on the calibration data set with full cross-validation. The absolute value of β indicates the importance of the corresponding wavelength. Wavelengths with large β values (irrespective of the sign) were the most influential [37]. Various studies have reported using WRC for key wavelength selection in different applications of HSI. For example, HSI data of coffee beans was used to determine the caffeine content and 12 important wavelengths were extracted [38]. Weighted regression analysis was performed to recognise 6, 24, and 15 important wavelengths for colour, pH, and tenderness prediction of beef slices, respectively [39].

## 3. Results

### 3.1. Full-Range PLS-DA Model

**Figure 3:**
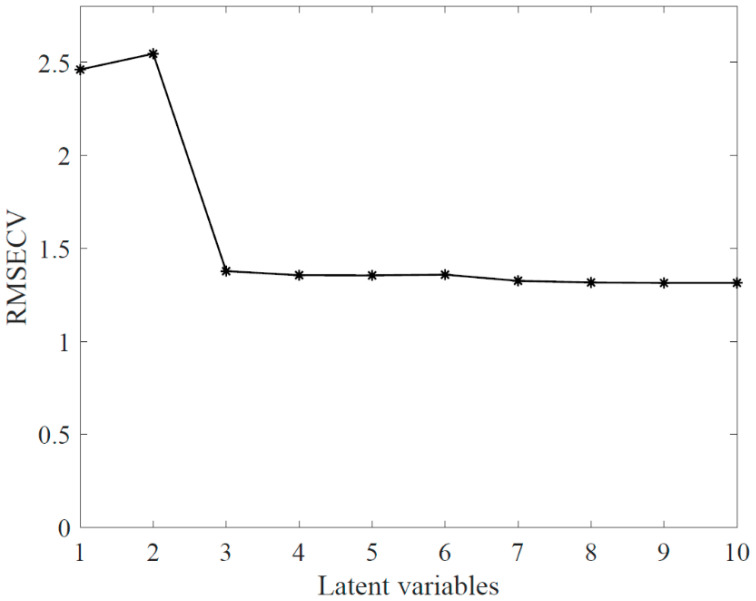
Latent variable selection using the root mean square error of cross-validation.

One thousand spectra were randomly selected from every single hyperspectral image used in the model. For predicting milk powder particle size classes, the discriminant analysis was performed by the PLS method using spectra obtained from the three size fractions (coarse (C), medium (M), and fine (F)). This model was trained (i.e., calibrated) and validated with the milk powder size fractions of different batches. As mentioned earlier, the samples from seven batches were used for the model calibration and the samples from the other three batches were used for the model validation. Figure 3 shows that the parsimonious number of latent variables (LV) was three as more than three LV did not significantly reduce RMSECV.

**Figure 4:**
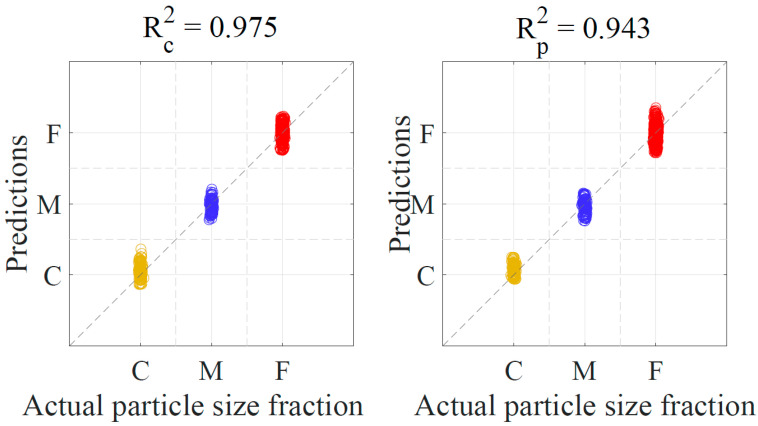
Prediction of milk powder samples into coarse, medium, and fine fractions.

Spectral pre-treatments are usually applied prior to developing the PLS-DA model. Pre-treatments including differentiation, and smoothing were tested before the data analysis, and smoothing showed improvements compared to taking derivatives of the spectra. Therefore, smoothing was applied to all spectra of milk powder samples before the PLS-DA model. An equal number of pixel spectra were selected from the hyperspectral images of the coarse, medium, and fine milk powder samples. Each point on the Figure 4 represents a single spectrum. These spectra and their corresponding class ‘dummy’ variable 1, 2, or 3 (represents C, M, or F, respectively) were involved to build the calibration and prediction model. Spectra whose predicted ‘dummy’ variable was within the range of 0.5–1.5 was classified as coarse particle fraction ‘C’. Similarly, the spectrum of predicted variables of 1.5–2.5, and 2.5–3.5 were classified as medium particle fraction ‘M’ and fine particle fraction ‘F’, respectively. A standard deviation of 0.015, in the form of a Gaussian distributed noise source, was added to the abscissa to show the spread of the data. It was observed that the PLS-DA model can successfully predict the milk powder particle size fractions using the spectral data, as the correlation coefficient values for the calibration and prediction of the PLS model were ≥0.94 (as shown in Figure 4). Classification accuracy, sensitivity, and specificity for selected spectrum from samples of individual particle size fractions have been presented in discussion section. However, it is worth mentioning that all the 27 samples of the validation set of discrete particle size fractions were correctly classified to their respective class C, M, or F.

### 3.2. Selection of Wavelengths

#### 3.2.1. Reduced Number of Wavelengths Based upon Principal Component Analysis (PCA)

After pre-processing by spectral smoothing and noise reduction, PCA was performed on the spectra of three size fractions of milk powder, and hence a large amount of spectral information of each pixel was represented by three PCs.

**Figure 5:**
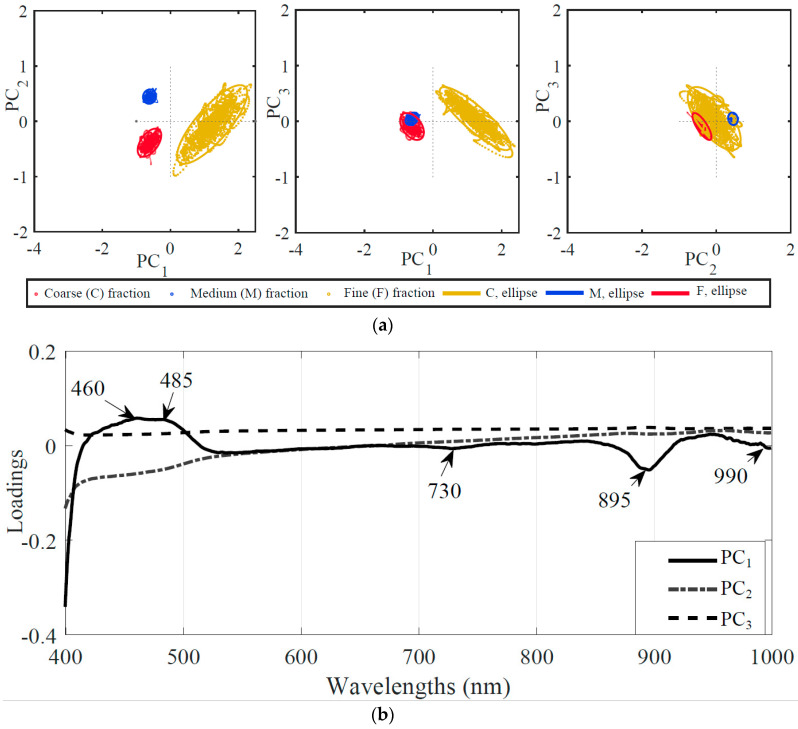
Principal component analysis of coarse, medium, and fine fraction of milk powder samples. (**a**) score plots; (**b**) loading plots.

The score and loading plots of the PCA are presented in Figure 5. Different colours were used to represent pixel spectra of the different particle size fractions of milk powder. An ellipse was drawn across each cluster to present the 95% probability of occurrence of the sample within the confined area. The score plots show the three PCs (PC_1_, PC_2_, and PC_3_) that comprised approximately 75% variance of all the spectral data of the three milk powder fractions. Almost 73% variance was associated with PC_1_ and PC_2_. The score plots differentiated the three milk powder fractions clearly across PC_1_ and PC_2_. The spectral information of various pixels of samples was presented as one single point on the PCA score plot.

The loading plot in a PCA shows the explanatory strength of the PCs towards the original data set’s wavelength variable. The wavelengths having high loading values (regardless of sign) are good candidates to be effective wavelengths [40]. The loading plot also indicated that PC_1_ was most influenced by wavelengths in the 400–550 nm and 850–1000 nm regions, while PC_2_ was also affected by the 400–550 nm wavelengths. The third principal component PC_3_ seemed least related across the whole wavelength range. With three PCs, at least three original wavelengths were needed for re-modelling the data. Therefore, loading vectors of the three PC’s plotted over the complete spectral range and it was observed that at least five local minima and maxima on PC_1_ and PC_2_ could be obtained, as shown in Figure 5 Wavebands of these minima and maxima were important to the clustering analysis in PCA [41]. Therefore, centred wavelengths within these minima and maxima regions from the loading curve were chosen. According to the loadings of PC_1_ to PC_3_ (Figure 5), two wavelengths of 460 and 485 nm were selected mutually from PC_1_ and PC_2,_ and a further three wavelengths of 730, 895 and 990 nm were selected from PC_1_, respectively. A model developed with these five wavelengths was named as PCA-PLS-DA.

#### 3.2.2. Reduced Number of Wavelengths Based upon Weighted Regression Coefficient (WRC)

**Figure 6:**
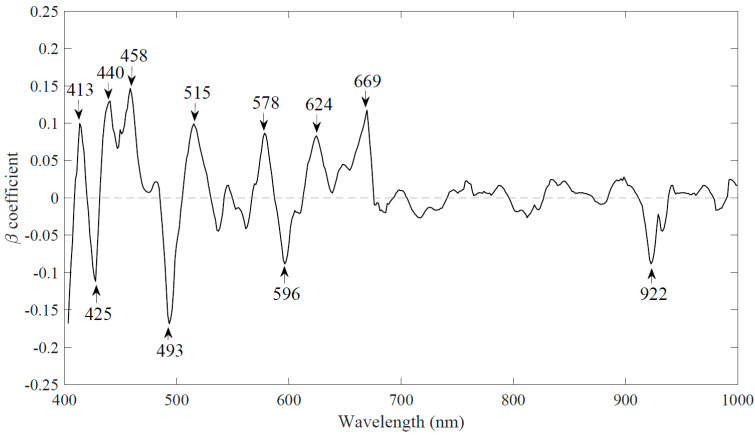
Selection of eleven wavelengths from the weighted regression coefficient method.

This technique reduced the number of wavelengths by selecting only those wavelengths that highly contribute toward the regression coefficient β. For this purpose, the spectral data was adjusted to the same scale. Afterwards, weighted regression coefficient β was plotted for entire spectral range, as shown in Figure 6. Higher values of β reflected the significance of the wavelengths. However, no particular threshold value was selected. Peak values of the regression coefficient β plot could be chosen [42].A set of 11 wavelengths was identified. These wavelengths were used to replace the full spectrum to build a simplified PLS-DA model. This model was named as WRC-PLS-DA. These wavelengths were 413, 425, 440, 458, 493, 515, 578, 596, 624, 669, and 922 nm. It is interesting that 10 out of 11 wavelengths belong to the visible region of the spectrum. This confirms the importance of the visible region in the multivariate data analysis of the milk powder samples with varying size particles.

## 4. Discussion

Notwithstanding the encouraging results found using the full wavelengths model, it is beneficial to use only a few variables for accurate, simplified, and robust classifications from hyperspectral data [43]. In Section 3.2., five wavelengths were selected by PCA loading analysis and 11 wavelengths were identified by the weighted regression coefficient technique. Simplified PLS-DA models with a reduced number of wavelengths were developed. Table 1 shows a comparison of models in terms of calibration performance (i.e., R_c_^2^, RMSEC, R_cv_^2^, and RMSECV), prediction performance (i.e., R_p_^2^ and RMSEP), residual predictive deviation (RPD), and computation time. Computation time was recorded as execution time for model to produce a results data set by an Intel Core i7 CPU with the dual-processor running at 2.60 GHz and 2.10 GHz with a memory capacity of 16 GB. Models were run 20 times and execution time was recorded. The average execution time is presented in Table 1 Fast computation was observed for the reduced wavelength models as it took 2.13 s on average for the model using five wavelengths identified by PCA loadings, while 2.82 s was the average execution time with 11 wavelengths of WRC-PLS-DA model for the classification of three individual fractions of milk powder. However, a model built with full spectral information of milk powder fractions was taking more than 30 s on average to produce results.

The regression coefficient R_p_^2^ was slightly improved from 0.943 of PLS-DA model to 0.962 for PCA-PLS-DA. The best regression coefficient R_p_^2^ was for the WRC-PLS-DA model with 0.979. However, significant differences were observed when PLS-DA models were evaluated by RMSEP and computational time. Root mean square error was reduced from 0.142 for a complete spectrum model to 0.066 for the model that was based upon reduced wavelength derived from PCA and 0.013 for model built with wavelengths selected by PCA. In terms of R_p_^2^ and RMSEP, WRC-PLS-DA resulted in better performance. The prediction performance of WRC-PLS-DA was better than the other models in terms of R_p_^2^ and RMSEP. Residual predictive deviation for all models was greater than five which indicates satisfactory performance of all models.

Performance of these models were evaluated for their respective classification accuracy as well (as presented in Table 2). Classification performance indicators such as sensitivity, specificity and overall accuracy of the model were calculated [44]. Prediction of class from a thousand spectra taken from single samples of milk powder of three classes—coarse, medium, or fine—were analysed. There were a total 21 samples in duplicate (i.e., 2 × 21 × 1000 spectra of each particle size fraction). Green cells in the Table 2 show the number of true positives (TP) of each particle size class e.g., a spectrum extracted from a coarse particle fraction of milk powder samples was predicted to be class ‘C’. False negative (FN) prediction was also estimated for the three particle size classes. A false negative number is the number of spectra that were assigned to an incorrect class and showed as red cells in Table 2. Furthermore, sensitivity and specificity of each class were also calculated. Sensitivity determined the ratio of the true positive prediction number to total number of spectra in a respective particle size class. It is noteworthy that the WRC-PLS-DA model showed the highest sensitivities of 0.964, 0.877 and 0.924 for coarse, medium, and fine particle size classes, respectively. Similarly, specificity referred to the actual negative prediction number ratioed to the total number of spectra that were not part of a respective class. It was observed that the coarse particle fraction had a greater than 96% probability for not being misclassified. A lower specificity may show a higher chance of predicting a false positive for the medium and fine particle spectra in these classification models. The highest overall accuracy in these three models was observed for WRC-PLS-DA i.e., 92.2%. Notwithstanding the similar predictive performance of these models, their classification performance indicators were distinctive.

**Figure 7:**
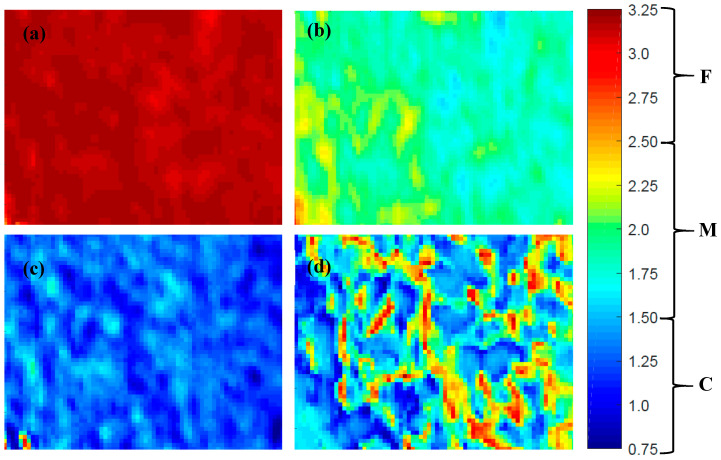
Example of prediction map of discrete particle fractions of milk powder (**a**) fine particle fraction sample; (**b**) medium particle fraction sample; (**c**) coarse particle fraction sample; and (**d**) recombined fractions sample.

The prediction maps of three discrete particle size samples and one recombined milk powder sample are shown in Figure 7. These maps present the classification of spatial pixels of a milk powder sample to their predicted particle size class. These maps show a clear visual discrimination between the coarse, medium, and fine particle size fractions. A reference scale is also presented here to refer to the range of predicted ‘dummy’ variable and its respective class of PLS-DA model. The result from the recombined milk powder sample suggests the feasibility of using hyperspectral imaging to visualise milk powder samples having varied particle size fractions. However, an even more comprehensive study with a large set of milk powder samples with varying particle sizes could be helpful for industrial applications where milk powder particle size affects the physical and functional properties of the final product.

## 5. Conclusions

This research was conducted to explore the potential suitability of visible and near-infrared (400–1000 nm) HSI for classifying three discrete particle size fractions of milk powder. Based on full spectral wavelengths, the PLS-DA model with R_p_^2^ of 0.943 was able to classify coarse, medium, and fine fractions of milk powder in 32 s on average. Important wavelengths identified by the PCA were five and by weighted regression coefficient were 11. Simplified PLS-DA models based upon these reduced numbers of wavelengths improved the prediction performance of the models. The WRC-PLS-DA model yielded better predictability with R_p_^2^ 0.98, RMSEP 0.013, RPD 5.94, and 92.2% classification accuracy. However, the fastest average computation time was recorded as 2.13 s for the PCA-PLS-DA model. Results of this preliminary study suggest that this method could be feasible to further investigate for rapid and non-invasive measurements of milk powder particle size for future at-line/on-line applications. It could also contribute to the design of future sensors by an initial choice of decisive wavelengths, allowing maximum discrimination between different particle size particles.

## Figures and Tables

**Table 1:** Regression performance of models for prediction of particle size fraction of milk powder.

Model	Wavelengths	LVs	Calibration		Prediction		Cross-Validation		RPD	Computation Time (s)
			R_c_^2^	RMSEC	R_p_^2^	RMSEP	R_cv_^2^	RMSECV		
PLS-DA	933	3	0.975	0.128	0.943	0.147	0.954	0.129	5.835	32.2 ± 1.5
WRC-PLS-DA	11	3	0.982	0.016	0.979	0.013	0.979	0.015	5.942	2.8 ± 0.3
PCA-PLS-DA	5	3	0.971	0.062	0.962	0.066	0.964	0.648	5.716	2.1 ± 0.2

**Table 2:** Classification performance of models for prediction of particle size fraction of milk powder.

PLS-DA		Predicted Class			FN	Sensitivity
		C	M	F		
Actual class	C	37,651	3964	385	4349	0.897
	M	1236	31,295	9469	10,705	0.745
	F	1061	5067	35,872	6128	0.854
FP		2297	9031	9854	Overall accuracy	0.832
Specificity		0.967	0.890	0.875		
WRC-PLS-DA		Predicted class			FN	Sensitivity
		C	M	F		
Actual class	C	40,511	1086	403	1489	0.964
	M	1909	36,854	3237	5146	0.877
	F	63	3112	38,825	3175	0.924
FP		1972	4198	3640	Overall accuracy	0.922
Specificity		0.975	0.949	0.955		
PCA-PLS-DA		Predicted class			FN	Sensitivity
		C	M	F		
Actual class	C	38,456	2946	598	3544	0.916
	M	1380	35,591	5029	6409	0.847
	F	345	3047	38,608	3392	0.919
FP		1725	5993	5627	Overall accuracy	0.894
Specificity		0.977	0.928	0.929		

Whereas, C, M, and F respective class of coarse, medium, and fine particle fraction, FN false negative, FP false positive, Green cells TP true positive, and red cells false negative.
